# Regulation of mechanotransduction: Emerging roles for septins

**DOI:** 10.1002/cm.21485

**Published:** 2018-10-10

**Authors:** Maxine Lam, Fernando Calvo

**Affiliations:** ^1^ Tumour Microenvironment Team, Division of Cancer Biology Institute of Cancer Research London United Kingdom; ^2^ Tumour Microenvironment Team, Department of Molecular and Cellular Signalling, Instituto de Biomedicina y Biotecnología de Cantabria Santander Spain

**Keywords:** cytoskeleton, mechanobiology, septin

## Abstract

Cells exist in dynamic three‐dimensional environments where they experience variable mechanical forces due to their interaction with the extracellular matrix, neighbouring cells and physical stresses. The ability to constantly and rapidly alter cellular behaviour in response to the mechanical environment is therefore crucial for cell viability, tissue development and homeostasis. Mechanotransduction is the process whereby cells translate mechanical inputs into biochemical signals. These signals in turn adjust cell morphology and cellular functions as diverse as proliferation, differentiation, migration and apoptosis. Here, we provide an overview of the current understanding of mechanotransduction and how septins may participate in it, drawing on their architecture and localization, their ability to directly bind and modify actomyosin networks and membranes, and their associations with the nuclear envelope.

## 1 MECHANOTRANSDUCTION IN A CELL

Cells in living organisms are constantly subjected to a myriad of physical forces as a result of their physical interaction with other cells, the extracellular matrix (ECM), fluid flows or mechanical constrictions. Therefore, living cells have acquired exquisite mechanisms that enable them to constantly and rapidly respond to mechanical forces, with cellular responses as diverse as migration, proliferation, differentiation and apoptosis (DuFort, Paszek, & Weaver, 2011; Lecuit, Lenne, & Munro, 2011; Petridou, Spiró, & Heisenberg, 2017). Mechanotransduction is the process whereby cells sense changes to their physical environment and translate them into biochemical signals. These biochemical signals can take the form of cytoskeletal rearrangements affecting cellular and nuclear morphology or the activation of signalling cascades, all of which ultimately lead to changes in gene expression.

**Figure 1:**
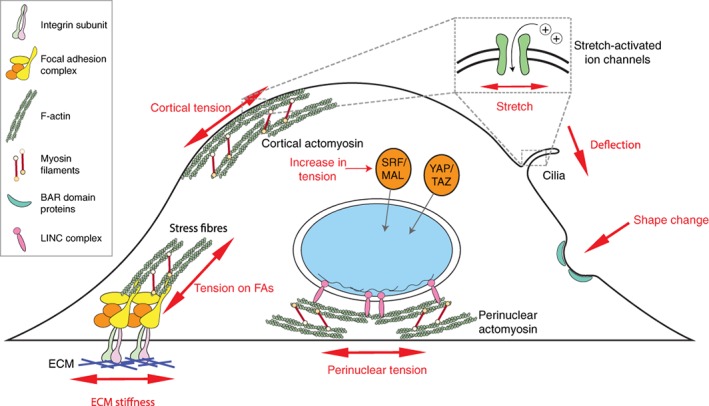
Mechanosensitive subcellular structures: Mechanical forces on cells (red font, arrows) are sensed by various subcellular proteins and complexes indicated in the diagram. At the cell surface, the actomyosin cortex coupled to the plasma membrane (PM) results in a contractile cell surface under tension. ECM stiffness is sensed by focal adhesion (FAs) complexes, via integrin subunits that transverse the cell surface and couple the ECM to the intracellular surface. Inside, stable FAs are associated with contractile actomyosin bundles that exert tension on the FA complex, and lead to further downstream signalling, including increasing cell contractility and nuclear translocation of transcription factors such as SRF/MAL and YAP/TAZ. Similarly, perinuclear actomyosin networks are connected to the nucleus via ‘linker of nucleoskeleton and cytoskeleton’ (LINC) complex and are able to directly transmit forces on the nucleus and modulate chromatin localization and gene transcription. At the PM, stretch‐activated ion channels modulate their permeability in response to changes in cell surface tension. Stretch‐activated ion channels are also localized at the base of non‐motile cilia, which are able to detect forces such as fluid shear force. Bin/Amphiphysin/Rvs (BAR) domain proteins localize to sites of membrane curvature, such as indentations or protrusions. Both stretch‐activated ion channels and BAR‐domain proteins have been shown to modulate signalling pathways and cytoskeletal rearrangements associated with mechanotransduction [Color figure can be viewed at wileyonlinelibrary.com]

Cells are able to sense changes to the physical environment through a range of mechanosensitive subcellular elements (Figure 1). These structures respond to forces in the form of protein conformational changes, changes in molecular interactions or localization. At the surface of the cell, large protein complexes like focal adhesions (FAs) link the ECM to the intracellular surface and cytoskeleton (Seetharaman & Etienne‐Manneville, 2018; Sun, Guo, & Fässler, 2016), while adherens junctions and tight junctions form between cells (Leckband & de Rooij, 2014). When these adhesion complexes are under tension, due to an increase in ECM stiffness or tissue tension, proteins such as vinculin and *α*‐actinin undergo conformational changes. This reveals cryptic binding sites that trigger signalling cascades that lead to the stabilisation and maturation of the adhesion complex, as well as the recruitment of contractile filamentous actin (F‐actin) bundles (Leckband & de Rooij, 2014; Sun et al., 2016). Additionally, stretch‐activated ion channels embedded within the plasma membrane (PM) can be directly modulated in response to changes in surface tension (Ranade, Syeda, & Patapoutian, 2015), while Bin/Amphiphysin/Rvs (BAR) domain proteins relocalize to sites of membrane curvature and deformation (Diz‐Muñoz, Fletcher, & Weiner, 2013; Vogel & Sheetz, 2006).

The actomyosin cytoskeleton in particular plays a critical role in mechanotransduction, acting both as a global mechanosensor and an essential relay for signal transduction. It interacts with almost all the previously mentioned mechanosensing components (Fletcher & Mullins, 2010; Iskratsch, Wolfenson, & Sheetz, 2014; Ohashi, Fujiwara, & Mizuno, 2017 March 1; Petridou et al., 2017), and reorganizes in response to changes in cell shape and tension (Ohashi et al., 2017 March 1; Schiffhauer et al., 2016). Thus, as matrix stiffness or tension exerted on a cell increase, cells respond by reorganizing the cytoskeleton, generating actin stress fibres (SFs) and increasing cell contractility. This response is critical in cellular homeostasis as it harnesses and balances the mechanical forces exerted on adherent and migrating cells (Ohashi, Fujiwara and Mizuno, 2017 March 1). The ability of cells to reorganize their cytoskeleton and intrinsic cell mechanics is therefore a key element in mechanoresponse and mechanotransduction.

Importantly, all these structures not only sense mechanical stimuli but are able to trigger signalling cascades throughout the cell that overlap with classical signal transduction. One classical example is FA formation and the activation of Src and FA kinases FAK in response to matrix stiffness, which leads to modulation of a myriad of signalling networks including the RhoA pathway. RhoA in turn propagates the signal by promoting actin remodelling and contractility, inducing protein phosphorylations, and altering the activity of signalling nodes and cellular processes (Brunton, MacPherson, & Frame, 2004). Ultimately, force‐dependent signalling can also affect the nuclear localization and function of transcriptional regulators such as SRF/MAL (Muehlich, Hermanns, Meier, Kircher, & Gudermann, 2016; Olson & Nordheim, 2010) and YAP/TAZ (Dupont et al., 2011), and the activation of force‐dependent transcription programs. In addition, the actomyosin cytoskeleton can serve as a physical link between mechanosensors at the cell surface and the nucleus via perinuclear actomyosin networks (Cho, Irianto, & Discher, 2017; Tajik et al., 2016). These structures connect the cytoskeleton to the nuclear lamina via the ‘linker of nucleoskeleton and cytoskeleton’ (LINC) complex, and together modulate nuclear architecture and force‐dependent gene expression or chromatin reorganization, which lead to changes in cell behaviour and even cell fate (Kirby & Lammerding, 2018; Uhler & Shivashankar, 2017).

## 2 SEPTINS: A NEW CYTOSKELETAL COMPONENT

**Figure 2:**
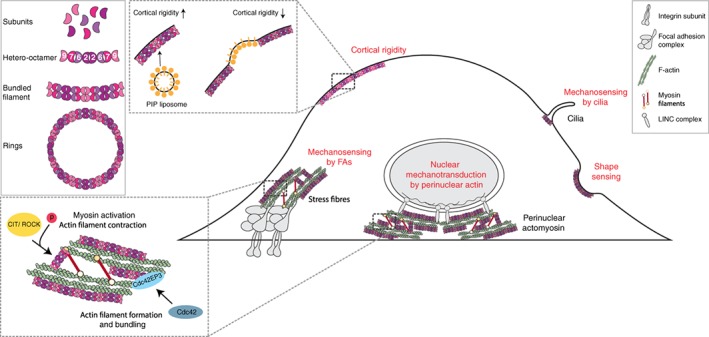
The interaction between septins, actin networks and mechanotransduction: Septins show preferential localization at sites that have a role in mechanotransduction (red font). Septins prominently colocalise with actin filaments within ventral stress fibres associated with FAs, as well as perinuclear actin. Septins promote the formation of contractile actomyosin networks, by binding to and promoting the recruitment of myosin to actin, as well as the activation of myosin by CIT and ROCK. Additionally, Cdc42 effector protein 3 (Cdc42EP3) binds to and activates septins, and Cdc42EP3 and septins promote the cross‐linking of actin bundles that promotes the formation of stable actin filaments. Septins are also able to directly affect cell surface tension by promoting the recruitment of PIP liposomes, and locally increasing membrane to reduce cortical tension and potentially affect the activity of stretch‐activated ion channels. Additionally, septins are found at the base of cilia, and are required for cilia formation. Hence, septins may be required for cilia mechanosensing through the formation of cilia, but also through the modulation of stretch‐activated ion channels found at the base of cilia. Septins have recently been found to relocalise to micron‐level membrane deformations, suggesting that they might sense changes in cell shape directly, similar to BAR domain proteins. *Inset: Septin filament formation of higher structures. Septin subunits form non‐polar palindromic heteroligomers that join end to end to form filaments. Septin filaments have a slight curvature, such that polymerization results in long curved filaments or rings* [Color figure can be viewed at wileyonlinelibrary.com]

Septins are a large family of guanosine triphosphate (GTP)‐binding proteins that are evolutionarily and structurally related to the Ras guanosine triphosphatase (GTPases) (Leipe, Wolf, Koonin, & Aravind, 2002). All septin proteins contain a conserved GTP‐binding domain, and N‐terminal proline‐rich and C‐terminal coiled coil domains that vary between family members (Barral & Kinoshita, 2008). In humans, there are 14 different septin genes that encode multiple isoforms. On the basis of homology in the proline‐rich and coiled‐coil regions, mammalian septins are categorized into four groups: SEPT2, SEPT6, SEPT7 and SEPT9. Unlike the monomeric small GTPases, septins can self‐assemble linearly into oligomers and polymers (Sirajuddin et al., 2007; Weirich, Erzberger, & Barral, 2008). Septin polymers consist of heterogeneous subunits where the basic septin oligomer is a hetero‐octamer composed of septins from each of the 4 groups. These octamers are typically arranged in a specific pattern (SEPT9–SEPT7–SEPT6–SEPT2–SEPT2–SEPT6–SEPT7–SEPT9) (Sellin, Sandblad, Stenmark, & Gullberg, 2011), although diverse hexamers and tetramers have also been described in some cells and tissues. Septin subunits polymerize into higher‐order structures forming linear and curved filaments, rings and meshworks (Makoto Kinoshita, 2003). End‐to‐end binding of septin hetero‐octamers results in nonpolar filaments (Makoto Kinoshita, 2003; Kinoshita, Field, Coughlin, Straight, & Mitchison, 2002), and lateral stacking of septin filaments and adaptor proteins leads to the formation of septin bundles (de Almeida Marques et al., 2012; Makoto Kinoshita, 2003) (Figure 2 **, inset**).

Septin network dynamics and organization are modulated by septin synthesis, degradation and post‐translational modifications such as phosphorylation, acetylation and SUMOylation, which can affect heteropolymer formation or the assembly of septins into higher‐order structures (Chahwan, Gravel, Matsusaka, & Jackson, 2013; Hernández‐Rodríguez & Momany, 2012; Ribet et al., 2017). In addition, septin filament formation is significantly influenced by septin association with actin and the PM. Both direct and indirect (via adaptor proteins) interaction with actin has been shown to promote the formation and bundling of septin filaments (Dolat et al., 2014; Farrugia & Calvo, 2016b; Joo, Surka, & Trimble, 2007; Makoto Kinoshita et al., 2002; Mavrakis et al., 2014; Smith et al., 2015), while actin depolymerization results in loss of septin filaments and the formation of septin rings (Makoto Kinoshita et al., 2002). At the PM, phosphatidylinositol‐4,5‐bisphosphate (PIP_2_) promotes the assembly of septin filaments (Tanaka‐Takiguchi, Kinoshita, & Takiguchi, 2009), and the sequestration or depletion of PIP_2_ results in the disruption of septin networks (Zhang et al., 1999).

## 3 THE ROLE OF SEPTINS IN ACTOMYOSIN CYTOSKELETON ORGANIZATION AND CELL MECHANICS

Due to its filamentous appearance as well as their association with cellular membranes and actomyosin networks, septins have been increasingly recognized as unconventional cytoskeletal components (Mostowy & Cossart, 2012). Additionally, because of the frequent colocalization of septins and actin, it is often suggested that septins regulate actin, or vice versa (Elias T Spiliotis, 2018). In non‐dividing cells, septins localize particularly along ventral SFs (Calvo et al., 2015; Dolat et al., 2014; Joo et al., 2007; Makoto Kinoshita et al., 2002; Kremer, Adang, & Macara, 2007) where they also form wavy filaments that connect nearby SFs (Calvo et al., 2015; Dolat et al., 2014). Underlying a functional role of septins in actin cytoskeleton regulation, silencing septin expression results in dramatic changes in cell shape and disruption of ventral SFs (Calvo et al., 2015; Dolat et al., 2014; Schmidt & Nichols, 2004).

In many cellular systems, septins are also particularly enriched in the perinuclear area where they form a dense network of filaments that colocalizes with actin and myosin‐II fibres (Calvo et al., 2015; Makoto Kinoshita et al., 2002; Schmidt & Nichols, 2004; Verdier‐Pinard et al., 2017). Recent reports suggest that septins are actively involved in the generation of these structures, as disrupting septin expression negatively affects the integrity of the perinuclear actin network (Calvo et al., 2015; Farrugia & Calvo, 2016a; Liu, Vong, Liu, & Zheng, 2014; Verdier‐Pinard et al., 2017).

Septins are also prevalent in contractile actin rings, such as those responsible for cellularization in the *Drosophila* embryo (Mavrakis et al., 2014) or the cytokinetic ring of mitotic cells (Elias T Spiliotis, 2018), where they aid in actin organization. *Drosophila pnut* (human SEPT7) promotes the formation of well‐bundled actin rings to ensure efficient contraction of the actomyosin ring (Mavrakis et al., 2014), while loss of septins in mitotic cells generally leads to defects in cytokinetic furrow ingression (Joo et al., 2007; M Kinoshita et al., 1997; E T Spiliotis, 2005) or abscission (Estey, Di Ciano‐Oliveira, Froese, Bejide, & Trimble, 2010; Surka, Tsang, & Trimble, 2002).

Several mechanisms have been proposed whereby septins promote actin filament formation. SEPT9 has been shown to protect nascent actin filaments from depolymerizing forces by competing with the actin‐severing protein cofilin, for binding with actin (Dolat et al., 2014; Smith et al., 2015). Alternatively, septins can regulate the localization and activity of the adaptor protein NCK, which is involved in the coordination of FA signalling and actin cytoskeleton (Kremer et al., 2007). Septin complexes bundle actin filaments *in vitro* (Makoto Kinoshita et al., 2002; Mavrakis et al., 2014) and SEPT2 − SEPT6 − SEPT7 complexes appear to promote the formation of long, curved actin filaments that are coated with septins (Mavrakis et al., 2014). Interestingly, the linear or curved morphology of actin depends on the filamentous state of septins, suggesting that higher‐order septin filaments may provide a template for the linear polymerization of actin.

Alternatively, septins may regulate actin organization by modulating or participating in Rho GTPase signalling. SEPT9 has been shown to bind directly to a Rho‐guanine nucleotide exchange factor (GEF) (ARHGEF18), via its N‐terminal domain, thereby inhibiting RhoA signalling and actin SF formation (Nagata & Inagaki, 2004). Additionally, another Rho GTPase, Cdc42, has been shown to affect the localization of septins, and this effect is mediated by the BORG family of Cdc42 effector proteins or Cdc42EPs (Farrugia & Calvo, 2016b). Cdc42EPs directly bind to and regulate septins (Joberty et al., 2001; Sheffield et al., 2003), and are in turn regulated by Cdc42 activity (Farrugia & Calvo, 2016a; Hirsch, Pirone, & Burbelo, 2001; Joberty, Perlungher, & Macara, 1999; Shlomi et al., 2017). Thus, Cdc42 binding is required for Cdc42EP3 to promote the formation of septin filaments and actin SFs (Farrugia & Calvo, 2016a), possibly acting like a molecular bridge reinforcing the connections between septin and actin filaments (Calvo et al., 2015).

Septins also influence actin organization and contractility by directly associating with myosin‐II structures. Myosin‐II interacts with SEPT2 through the coiled‐coil domain of its heavy chain and therefore can serve as an adaptor protein linking septin filaments with actin microfilaments (Joo et al., 2007). Disruption of the SEPT2‐myosin II interaction results in loss of SFs in interphase cells, and incomplete cytokinesis during mitosis. Additionally, SEPT2 may also provide a scaffold for the phosphorylation of myosin‐II light chain by the citron Rho‐interacting kinase (CIT) and the Rho‐associated protein kinase (ROCK), which stimulates myosin‐II contractility (Joo et al., 2007).

Besides their effect on actomyosin networks, membrane‐bound septins can assemble in arrays on the cytoplasmic leaflet of membrane bilayers and dramatically influence their shapes directly (Tanaka‐Takiguchi et al., 2009). This septin meshwork generates a curved, rigid surface with high affinity for PIP‐ and PIP_2_‐containing liposomes, thus sequestering excess membrane. This process can influence local PM tension and induce the formation of long tubules in vitro.

Unsurprisingly, through their interactions with the actomyosin cortex and the PM, septins have been shown to stabilize the cell cortex and regulate cell surface tension. Thus, cells lacking SEPT2 or SEPT11 lose cortical elasticity to a similar degree as when F‐actin is reduced (Mostowy et al., 2011); septins stabilize the cell cortex of T lymphocytes (Gilden, Peck, Chen, & Krummel, 2012); and cells without septins experience dramatic membrane blebbing due to a soft and unstable cortex (Gilden et al., 2012; Tooley et al., 2008).

## 4 SEPTINS IN MECHANOTRANSDUCTION

Septin filaments are less dynamic than F‐actin and do not have associated motor activity or “stretchable” domains that would enable them to exert forces or respond to mechanical cues, the way that actomyosin networks or adhesion complexes do. However, because of their functional interaction with key mechanotransduction elements, evidence is emerging suggesting a potential role of septins in mechanobiology (Calvo et al., 2015; Dolat et al., 2014; Simi et al., 2018). In addition, recent studies have revealed that septin organization itself is mechanically regulated, and that septins participate in the regulation of canonical mechanotransduction pathways.

While the interaction between septins and SFs had been observed before, Dolat et al. were the first to identify a relationship between septins, SF formation and FA maturation (Dolat et al., 2014). In transformed renal epithelial cells, SEPT9 crosslinks and organizes preassembled actin rings to promote SF formation, and septin depletion resulted in smaller and more transient and peripheral FAs, which ultimately perturbed cell motility. Because of the importance of SFs and FAs in mechanosensing and mechanotransduction, this hinted strongly at a potential role for septins in these processes. This hypothesis was recently confirmed using cancer‐associated fibroblasts (CAFs). CAFs are fibroblasts generally found in solid tumours that present a pathologically activated phenotype that enables them to generate environments for cancer cells to propagate and acquire aggressive phenotypes (Kalluri, 2016). CAFs are much more mechanosensitive than normal fibroblasts, and their tumorigenic properties are in part due to their ability to alter their behaviour on stiff matrices (Calvo et al., 2013). Compared to normal fibroblasts, CAFs on stiff matrices generate enhanced actomyosin SFs, promoting FA maturation, Src and FAK signalling, and activation of the mechanotransducer transcription factor YAP (Calvo et al., 2015). This heightened mechanosensitivity is a direct consequence of the upregulation of septin regulator Cdc42EP3 in CAFs, which directly promotes the formation of SEPT2 and SEPT7 filamentous structures in response to increased matrix stiffness. Importantly, loss of Cdc42EP3, SEPT2 or SEPT7 leads to reduced mechanoresponses to matrix stiffness (i.e., reduced SFs, Src/FAK signalling and YAP activation), and subsequent decrease in the mechanical and tumorigenic properties of CAFs.

This study provides landmark evidence of the mechanical regulation of septin architecture and their role in mechanotransduction, and it is tempting to speculate that these findings might be extensible to other contexts where similar activities have been reported. In highly mechanosensitive mouse cardiac endothelial cells (Hahn & Schwartz, 2009), where septins associate with Cdc42EP1, both are required for persistent directional migration and angiogenesis. This function was associated to a positive role of Cdc42EP1 and septins in the formation of perinuclear actomyosin fibres (Hahn & Schwartz, 2009). Perinuclear actin networks are important for mechanosensing and mechanotransduction to the nucleus, through their association with the LINC complex and highly tensile perinuclear adhesions, which lead to downstream YAP and SRF/MAL nuclear translocation (Ho, Jaalouk, Vartiainen, & Lammerding, 2013; Kim, Chambliss, & Wirtz, 2013; Shiu, Aires, Lin, & Vogel, 2018). Interestingly, Cdc42EP1 as well as both YAP and SRF/MAL have been shown to be critical for cardiac development (Liu et al., 2014; Parlakian et al., 2004; Xin et al., 2013). Whether cardiac defects after Cdc42EP1 deletion are associated with perinuclear actin disruption leading to defective mechanotransduction via YAP or SRF/MAL is a possibility that warrants further investigation. Still to be determined is whether septins directly associate with the LINC complex, and whether they can effect and respond to changes in nuclear stiffness or architecture the same way that actomyosin networks do. To begin, better characterization of nuclear architecture and morphology, and associated changes in epigenetic and gene expression programs after septin perturbation are required. It may be possible that, similar to its input in FA maturation (Calvo et al., 2015; Dolat et al., 2014), septin filaments are only indirectly associated to LINC function via actin, and that they participate in this process solely by reinforcing perinuclear actomyosin fibres.

Mechanical regulation of septins has since also been shown in other cellular contexts. In the mammary epithelium, cells that have undergone epithelial‐to‐mesenchymal transition (EMT), display increased mechanosensitivity, with cells failing to resolve the final stage of cytokinesis on stiff matrices but not on soft matrices (Simi et al., 2018). On stiff matrices, there is a force‐dependent upregulation of the transcription factor Snail in cells that have undergone EMT, which directly promotes SEPT6 expression (Simi et al., 2018). Mechanistically, SEPT6 upregulation results in its persistence in the midbody, leading to failure of midbody resolution and multinucleated cells (Simi et al., 2018). Yet, it is still unclear whether SEPT6 acts in a similar manner to regulate mechanosensitive abscission in other cell types, and whether other septins operate in a similar manner. Noteworthy, in this system SEPT6 appears to function as a dominant negative factor to block exocyst delivery, an activity previously described in other septin isoforms (i.e.,SEPT9_ i4) (Estey et al., 2010).

However, not all septins are upregulated with increasing matrix stiffness. In endothelial cells, *α*_*v*_ *β*_3_ integrin activation in response to matrix stiffness inhibits *SEPT9* expression, promoting cell proliferation (Yeh et al., 2012). At a molecular level, it was shown that *α*_*v*_ *β*_3_ integrin activation releases SEPT9‐bound ARHGEF18 leading to activation of RhoA, Src and Vav2 signalling as well as cell cycle progression (Nagata & Inagaki, 2004; Yeh et al., 2012). Interestingly, SEPT9 interacts with ARHGEF18 at its N‐terminal domain (Nagata & Inagaki, 2004), and depending on the presence of the domain, isoforms of SEPT9 have been shown to affect cell behaviour very differently in similar mechanical conditions (Connolly et al., 2014; Estey et al., 2010; Nagata & Inagaki, 2004; Verdier‐Pinard et al., 2017). This leaves open the question of whether SEPT9 isoforms are therefore differentially regulated in response to mechanical stimulus.

Besides regulating actomyosin organization, septins may also directly participate in mechanotransduction by their role in cell shape sensing. Septins have recently been shown to be able to sense membrane curvature at the micron‐scale and may serve as landmarks for eukaryotic cells to detect changes in cell shape (Bridges, Jentzsch, Oakes, Occhipinti, & Gladfelter, 2016). This function appears very similar to BAR‐domain proteins, which have been shown to modulate signalling pathways and cytoskeletal rearrangements associated with mechanotransduction (Diz‐Muñoz et al., 2013; Galic et al., 2012; Vogel & Sheetz, 2006). It is also possible that upon relocalizing to regions of cortical deformation (such as blebs or sites of mechanical perturbation), septins create locally distinct signalling platforms with their binding partners in the actomyosin network or within the phospholipid bilayer to coordinate a local response. In this way, septins would act as novel sensors of shape changes and simultaneously act as mechanotransducers through their interactions.

Additionally, septins are particularly enriched in cellular structures with high curvature that generate or are exposed to mechanical stress such as the contractile cytokinetic ring, the annulus of spermatozoa flagella, the base of protrusions such as cilia and dendrites, and the phagocytic cup formed during bacterial infection (Mostowy & Cossart, 2012). This is likely because septins are able to generate and stabilize curved cellular structures through their ability to promote the formation of actin filaments and locally rigidify the PM (Sirajuddin et al., 2007; Tanaka‐Takiguchi et al., 2009). By regulating PM curvature and tension, septins are likely to also affect the conformation of stretch‐sensitive ion channels, thus potentially modulating the cellular response to external stretch and downstream mechanotransduction (Pardo‐Pastor et al., 2018; Coste et al., 2010). This may be particularly important for cilia, which are specialized structures at the cell surface implicated in mechanosensing (Hoey, Downs, & Jacobs, 2012; Nauli et al., 2013). Primary cilia act as cellular antennas in which mechanical deflection by fluid flow or tissue deformation results in the opening of associated stretch‐activated channels at the base, and downstream signalling (Nauli et al., 2013; Spasic & Jacobs, 2017). Importantly, septins are required for the formation and maintenance of the primary cilium by controlling the localization of ciliary membrane proteins through their interactions with PM proteins (Palander, El‐Zeiry, & Trimble, 2017).

However, it remains unknown whether septins participate in mechanosensing and mechanotransduction at stretch‐activated channels and cilia. Future work will therefore require experiments directly testing the role septins in relaying mechanical cues picked up by cilia or PM deformation. This may require experiments directly studying the effect of septins on ion channel conformation and downstream signalling (such as Ca^2+^ concentrations), as well as measurements of PM tension with different septin network conformations.

## 5 CONCLUSIONS AND FUTURE PERSPECTIVES

Since their discovery, septins have rapidly emerged as important components of the cytoskeleton and PM. Now, there is increasing evidence that they have a strong influence on cell shape and contractility, through a large variety of functional associations with proteins in the actomyosin networks and PM (Figure 2). The actomyosin network in particular is an important structure in mechanotransduction, and septins have been found to be part of the cellular response to mechanical cues through their ability to modulate actomyosin structures. However, the study of septin‐actomyosin functional interactions in the context of mechanotransduction remains far from comprehensive. In addition, a role for septin‐dependent mechanotransduction at the level of PM curvature‐sensing or ion channel activity remains theoretical. Clearly, much more work needs to be done to determine whether septin function is directly influenced by mechanical forces and to identify effectors and activators of septin activity in the context of mechanotransduction. A crucial point will be to ascertain whether septin‐dependent rearrangements in actomyosin networks (including perinuclear architecture) and at the PM are associated with changes in mechanotransduction signalling and functions.

In addition, it would be interesting to determine whether other septin‐dependent signalling pathways that have not been associated with mechanotransduction may in fact be involved. SEPT9 isoform 1 (SEPT9_i1) interacts with HIF1*α* and increases its protein stability and transcriptional activity (Amir, Wang, Matzkin, Simons, & Mabjeesh, 2006). This interaction is dependent on SEPT9_i1 relocalization to the nucleus via importing‐*α* (Golan & Mabjeesh, 2013). Therefore, it may be speculated that processes affecting SEPT9_i1 localization and availability, such as force‐dependent septin relocalization or filament formation, may affect HIF1*α* activity.

Considering the effects of mechanical cues on cancer cell malignancy, cell differentiation and EMT, it is important to further study the role of septins in these processes from a mechanical perspective. Changes in septin expression have been observed in cancers, and septins have already been shown to be important for cancer cell invasion and survival (Angelis & Spiliotis, 2016). However, it is not known if septins are directly involved in modulating mechanotransduction pathways in this context, or if it is simply through septin‐dependent actomyosin regulation. In fibroblasts at least, septins appear to be dispensable for normal function but essential for CAF‐dependent promotion of a tumorigenic environment (Calvo et al., 2015), and targeting septin function may prove to be a unique method to perturb tumorigenic mechanotransduction pathways. Additionally, the induction of an EMT program in cells can increase septin expression (Dolat et al., 2014; Simi et al., 2018), and septins have been shown to be involved in EMT‐associated cell invasion (Dolat et al., 2014) and multinucleation (Simi et al., 2018). This suggests that septins might play a role in EMT, but it is still unclear if septin upregulation alone can affect the establishment of cell fate programs, or if they are activated downstream of EMT together with actomyosin reorganization to change cell behaviour.

These analyses will be complicated by the fact that cells contain several septins and septin isoforms, and that there is significant heterogeneity in the effects of septins and their isoforms on cellular behaviour (Connolly et al., 2011, 2014; Estey et al., 2010; Verdier‐Pinard et al., 2017). Furthermore, septin interaction partners are dependent on their assembly status (i.e., monomers, hetero‐oligomers or filaments), the type of structures they form (i.e., filaments, rings, meshworks) and their subcellular localization. The understanding of the role septins will likely be highly context‐dependent, and this is in line with their known ability to coordinate complex subcellular responses (Elias T Spiliotis, 2018).

Finally, classical mechanobiology techniques will be required to assess the role of septins in mechanotransduction. These include traction force analysis, and measurements of cell and nuclear shape and mechanical properties. In particular, it would be interesting to decipher the links between septins and key nodes in mechanotransduction such as FAs and LINC complexes. Are these interactions indirect through septin‐mediated actomyosin organization, or could septins play a direct role in signal transduction at these points?
